# Effects of Animal-Based and Plant-Based Nitrates and Nitrites on Human Health: Beyond Nitric Oxide Production

**DOI:** 10.3390/biom15020236

**Published:** 2025-02-07

**Authors:** Valentina Membrino, Alice Di Paolo, Tiziana Di Crescenzo, Monia Cecati, Sonila Alia, Arianna Vignini

**Affiliations:** 1 Department of Clinical Sciences, Università Politecnica delle Marche, 60100 Ancona, Italy; v.membrino@pm.univpm.it (V.M.); a.dipaolo@pm.univpm.it (A.D.P.); t.dicrescenzo@pm.univpm.it (T.D.C.); 2 Department of Human Sciences and Promotion of the Quality of Life, San Raffaele Roma Open University, 00166 Rome, Italy; moniacecati@gmail.com; 3 Research Center of Health Education and Health Promotion, Università Politecnica delle Marche, 60100 Ancona, Italy

**Keywords:** nitrite, nitrate, food additives, nitric oxide, plant-based food, animal-based food

## Abstract

Nitrate (NO_3_) and nitrite (NO_2_) are important nitrogen compounds that play a vital role in the nitrogen cycle, contributing to plant nutrition and broader ecological functions. Nitrates are produced from nitric acid (HNO_3_), while nitrites come from nitrous acid (HNO_2_). These substances are commonly found in the environment, especially in food and water, due to contamination from both human and natural sources. Human activities are major contributors to the high levels of nitrates found in water, leading to environmental pollution. Although nitrogen is crucial for plant growth, excessive fertilizer use has caused ecological disruptions. In plants, nitrates tend to accumulate primarily in the leaves of non-leguminous crops, such as leafy vegetables, which are known for their high nitrate content. Furthermore, nitrates and nitrites are added to animal-based foods, especially processed meats and cheeses, to prevent bacterial growth, slow spoilage, and improve flavor and color. The concentration of these compounds in food can vary due to different factors like farming practices, climate, soil conditions, and food production methods. This review seeks to examine the differences between the plant-based and animal-based sources of these compounds and assess their potential impact on human health, considering also the paradigm that goes beyond nitric oxide production.

## 1. Introduction

Nitrate (NO_3_) and nitrite (NO_2_) are important compounds composed of nitrogen and oxygen elements. They are formed as essential components of the nitrogen cycle, primarily due to the activity of nitrogen-fixing bacteria that thrive in the root systems of plants. As an integral part of this vital cycle, nitrates and nitrites are considered not only as nutrients but also as crucial sources of nitrogen for plants, as well as for the complex organisms that consume them. Specifically, nitrites are derived from nitrous acid (HNO_2_), whereas nitrates are produced from nitric acid (HNO_3_). This biochemical transformation underscores their significance in both plant nutrition and broader ecological interactions.

The levels of nitrates and nitrites are significantly elevated in the environment, making it quite easy to detect these compounds in several food products and water supplies. In addition, a variety of sources and processes can contribute to nitrate contamination across diverse ecosystems. For instance, an increased nitrate concentration in aquatic environments often results from nitrogen accumulation in the ecosystem, which can be attributed to both anthropogenic activities—such as agricultural runoff and industrial discharges—and geogenic sources, including natural geological processes. The main anthropogenic contributors to nitrate contamination include fertilizers, industrial nitrate application, waste from animals and humans, deforestation, landscaping, etc., [1]. However, the extensive use of inorganic fertilizers has resulted in a significant increase in nitrate concentrations in water resources across many parts of the world.

Nitrogen is an essential element required for plant chlorophyll synthesis, and this substantial demand for nitrogen to develop an effective photosynthetic system has created a significant necessity for nitrogen-based fertilizers, particularly among high-yielding crops [2]. The use of nitrogen fertilizers has risen significantly in recent years, especially in Asia [3], and this led to an improvement in global food production but, conversely, it has had a detrimental effect on the ecosystem. First of all, it causes soil and water pollution, and the final effect is that we can find nitrates and nitrites in drinking water and food. Nitrates in groundwater can lead to the contamination of drinking water, posing risks to both ecosystems and human health. In aquatic ecosystems, high nitrate levels contribute to eutrophication, where nutrient overload promotes algal blooms that deplete oxygen, creating “dead zones” that harm aquatic life. Furthermore, nitrate pollution disrupts biodiversity and alters the functioning of wetlands and riverine systems. Recent studies have emphasized that nitrate leaching is a persistent issue, with significant implications for long-term ecosystem health and sustainability [4]. Mitigating these environmental impacts requires the adoption of practices that reduce fertilizer runoff, such as precision agriculture and buffer zones around water bodies [5]. Indeed, high doses of nitrogen and/or nitrate fertilizers may lead to health issues. NO_3_-contaminated drinking water might be related to methemoglobinemia, thyroid conditions, diabetes, damaging reproductive outcomes, and cancer [6]. Plant-based food can supply a large intake of nitrogen compounds, especially food from vegetables that grow near the soil [7]. In non-leguminous crops, higher nitrate concentrations are typically found to accumulate mainly in the leaves, with comparatively lower levels in bulbs, seeds, fruits, roots, and tubers. Consequently, leafy vegetables like rocket, Swiss chard, spinach, lettuce, celery, and parsley are identified as key nitrate-accumulating species within the plant kingdom [8]. Moreover, the nitrate content in vegetables is significantly affected by seasonal variations and the cultivation methods used. The promise of improving biofortification methods to enhance the nutrient density of plants, including their nitrate levels, is significant. By focusing on the nitrate–nitrite conversion pathways, it is possible to develop crops that retain the health advantages of nitrate-derived nitric oxide and minimize the creation of harmful compounds [9].

However, vegetables are not the sole source of nitrates in the diet, as these compounds can also be found in animal-based products, where they are used to preserve processed meats and cheeses. Nitrites (sodium nitrite—E249 and potassium nitrite—E250) and nitrates (sodium nitrate—E251 and potassium nitrate—E252) are permitted as food additives by the European Union according to Commission Regulation (EU) No 1129/2011, which sets the maximum allowable amounts for their use in food processing [10]. Nitrates and nitrites are often incorporated into food products for several important purposes, including the inhibition of microbial growth (mainly Clostridium botulinum), the delay of rancidity, the enhancement of cured meat flavor and aroma, and the stabilization of meat’s appealing red color. The remarkable effectiveness of both nitrite and nitrate as food preservatives renders them indispensable additives in the meat industry, mostly for various cured meat products. Their ability to not only preserve but also enhance the sensory qualities of meat makes them crucial in food processing [11].

The intake of nitrates and nitrites from food can differ significantly between regions, primarily due to various influencing factors. These factors include agricultural practices, local climate conditions, the quality of the soil, the specific manufacturing processes employed, and the existing legislation on food production. Such variations can lead to considerable differences in the amount of these compounds available in the food sources across different geographical areas.

Very interesting are the data published by Bondonno et al., in a Danish Study. The research analyzed the dietary data of 52,247 participants over 24 years. The results indicated that a moderate to high intake of plant-based nitrates reduced overall mortality by 17%, particularly with regard to cardiovascular disease and cancer. In contrast, animal-based nitrates and products treated with nitrites were associated with higher mortality rates. The protective effect of plant-based nitrates was attributed to the formation of health-promoting nitric oxide [12].

It is also interesting to consider in this regard dietary habits in Mongolia, which are characterized by ultra-high consumption of red meat (300 g/day—more than 1700% compared to the boundary intake of 0–35 g/day established by the EAT-Lancet Commission within the Planetary Health Diet), and on the contrary, an adequate intake of fruits and vegetables of 8% (20 g/day) and 20% (73 g/day), respectively [13]. Such dietary patterns can lead the Mongolian population to malnutrition, micronutrient inadequacies, overweight and/or obese conditions, and related chronic diseases [14]. Healthy dietary patterns, such as the Mediterranean Diet or the Dietary Approaches to Stop Hypertension (DASH), have a dietary nitrate component—associated with fruit and vegetable intake [15]. Trials have demonstrated the beneficial impact of elevated levels of nitrate intake (400–800 mg/day) in particular on cardiometabolic and neurocognitive health [16,17]. A possible protective role on brain health may be attributed to dietary nitrate; however, the current knowledge is still lacking consistent evidence [18].

The French NutriNet-Santé cohort study, enrolling 101,056 adults, followed for 6.7 years, examined the association between the dietary intake of nitrites and nitrates, both from food additives and natural sources, and the risk of gastric cancer. The researchers found that high or moderate intake of nitrites was linked to an increased risk of gastric cancer. However, the evidence regarding the association between nitrite intake and other types of cancer was less consistent, indicating a need for further research to clarify these potential links [19].

A study in the United States highlighted increasing nitrate levels in water resources, linking nitrate ingestion from drinking water to adverse health effects, while noting that dietary nitrate ingestion is generally associated with beneficial health outcomes [20].

In 2020, the European Commission requested the European Food Safety Authority (EFSA) to re-evaluate all the food additives authorized prior to that year. As a part of this program, EFSA reassessed the acceptable daily intakes (ADIs) for nitrites and nitrates, specifically the sodium and potassium salts of nitrite (E 249 and E 250) and nitrate (E 251 and E 252). The current ADIs for nitrite, as recommended by EFSA, were published in June 2017 in two scientific opinions, and they reaffirm the same levels previously established by the European Commission’s former Scientific Committee for Food (SCF) in 1997 and the Joint FAO/WHO Expert Committee on Food Additives (JECFA) in 2002, which are 0.06 and 0.07 milligrams per kilogram of body weight per day (mg/kg bw/day), respectively. Instead, for nitrate, both committees set the ADI at 3.7 mg/kg bw/day [21].

These recommended ADIs for nitrites and nitrates are the same for adults, children, and infants because the safe doses are expressed in milligrams per kilogram of body weight; however, especially infants and children, due to their smaller body size, higher metabolic rate, and immature digestive systems, are proportionally more vulnerable to the harmful effects of these substances. Infants, especially those under 6 months of age, are at a significantly higher risk of experiencing the toxic effects associated with high nitrate consumption, which can lead to methemoglobinemia, commonly known as “blue baby syndrome.” This condition impairs the ability of red blood cells to carry oxygen and can be life-threatening in severe cases [22]. The ingestion of nitrites and nitrates is not limited to their presence as additives in food; it is also a consequence of their occurrence in drinking water. The World Health Organization (WHO) has set a maximum acceptable limit of 50 mg/L as NO_3_ or 11.3 mg/L NO_3_-N (multiply NO_3_ mg/L by 0.2258). This is in order to protect human health from the negative effects of nitrates, which include the risk of methemoglobinemia, as well as adverse pregnancy outcomes, cancer, thyroid disease and, according to a smaller number of studies, type 1 childhood diabetes (T1D), blood pressure, and acute respiratory tract infections [23].

Thus, the present review aimed to compare the differences between animal-based and plant-based nitrates and nitrites, and to summarize their effects on human health.

## 2. Chemical and Non-Chemical Nitrate Production Processes

It is essential to delineate the distinction between chemical and non-chemical nitrate production processes. The primary differentiation is based on the methodologies employed to synthesize nitrates.

The chemical method involves industrial processes that use chemical reactions to produce nitrates. The most common method is the Haber–Bosch process (for ammonia production), followed by the oxidation of ammonia with oxygen to produce nitric acid (HNO_3_). The nitric acid is then combined with a base (like ammonia) to form nitrate salts [24]. A popular example is ammonium nitrate (NH_4_NO_3_), commonly used in fertilizers and explosives. In this process, the nitrogen from the air is combined with hydrogen (from natural gas) to create ammonia, which is further processed to produce nitric acid and ultimately nitrates.

Non-chemical methods typically refer to natural processes or methods that do not involve complex chemical reactions. These can include biological nitrate formation and natural accumulation. Nitrates can be produced biologically through processes like nitrification, where bacteria in the soil or water convert ammonia (NH_3_) or ammonium (NH_4_⁺) into nitrate (NO_3_^−^). This is a key part of the nitrogen cycle in nature. Moreover, nitrates can also form through atmospheric processes, such as lightning, which can break down nitrogen molecules in the air and form nitrates that are deposited into the soil through rain [25].

## 3. Pathways of Nitric Oxide Formation: From Nitrate Ingestion to Health Implications

Once ingested through food and drinking water, nitrate is quickly absorbed by the stomach and small intestine and then enters the bloodstream [26]. After consumption, the plasma levels of nitrate remain high for 5–6 h. Then, a significant amount of the circulating nitrate is excreted by the kidneys, while up to 25% is actively absorbed, concentrated, and secreted into the saliva by the salivary glands [27]. In the oral cavity, commensal bacteria convert salivary nitrate to nitrite through the action of nitrate reductase enzymes [28]. These microorganisms occupy the crypts of the tongue and, in the absence of oxygen, utilize nitrate for respiration. The result is that nitrite bioavailability depends on the oral microbiota, and individual variations in its composition can influence nitrate reduction. Moreover, the use of mouthwash and antibiotics may disrupt the balance of the oral microbiota and, consequently, nitrite bioavailability.

**Figure 1:**
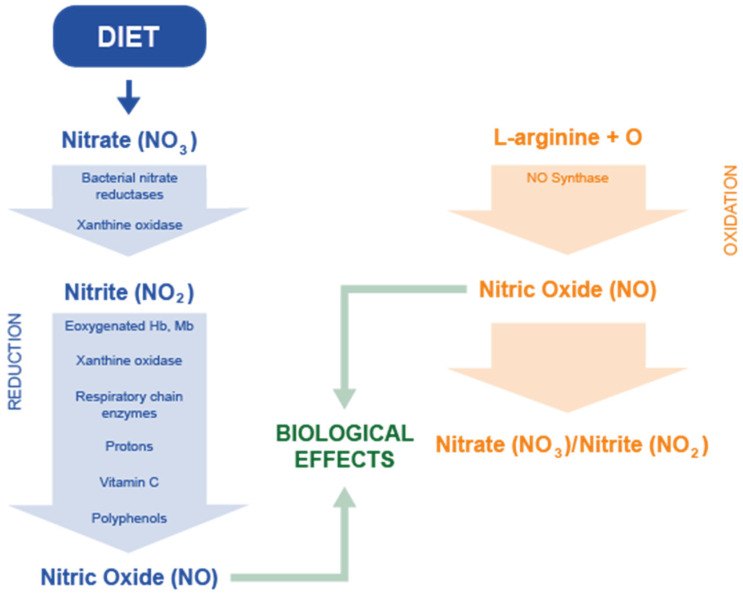
NO formation pathway.

Nitrite is swallowed, absorbed through the upper gastrointestinal tract, and released into the bloodstream. In the stomach and other gastric organs, the acidic environment can convert nitrite into nitric oxide (NO), an important cell signaling modulator. Additionally, circulating nitrite can be further transformed into NO through the enzymatic action of various enzymes with nitrite reductase activity (this refers to the exogenous production of NO, Figure 1). These enzymes are present in several types of cells, playing essential roles in the body’s physiological processes.

However, NO can also be produced endogenously via the L-arginine-NO pathway. The enzymes known as NO-synthases (NOSs) catalyze the conversion of L-arginine and molecular oxygen into NO. This NO is then quickly oxidized to form nitrite and nitrate; however, these compounds can be recycled, leading again to the formation of NO (Figure 1) [28]. NOS is the sole rate-limiting enzyme involved in NO synthesis from L-arginine. There are three isoenzymes of NOS: neuronal NOS (nNOS or NOS1), inducible NOS (iNOS or NOS2), and endothelial NOS (eNOS or NOS3). Under normal physiological conditions, nNOS and eNOS are the forms that are constitutively expressed, while under pathological conditions, iNOS is more likely to be produced [29].

NO possesses an extremely short half-life, typically lasting just milliseconds, whereas nitrite and nitrate have much longer half-lives, ranging from minutes to hours, and can be consistently measured in plasma.

In fact, the presence of nitrite could serve as a valuable marker for the formation of NO within the body.

NO is essential in regulating vasodilation, the process by which blood vessels widen. Additionally, it exerts an inhibitory effect on platelet aggregation, thereby enhancing overall blood circulation. Consequently, the body endogenously synthesizes NO to maintain and regulate a wide array of physiological functions, including blood pressure control, immune response, metabolism, and many other vital processes. This highlights the importance of NO in sustaining overall health and homeostasis [30].

Also, nitrate results are essential in maintaining homeostasis and mobilizing the body’s reserve potential, as well as in preventing diseases. In particular, dietary nitrates, contained in fruits and vegetables, are considered beneficial to human health as they promote microbiota homeostasis, regulate inflammation-immune homeostasis, and energy metabolism homeostasis, thus providing a multi-level homeostatic balance [31].

In the immune system, NO is vital for various antipathogenic and tumoricidal responses.

Paradoxically, it has also been implicated as a factor that can exacerbate the severity of different diseases, including cancer [32] and stroke [33].

The apparently contradictory effects of NO can be classified into two main types: direct effects and indirect effects.

Direct chemical reactions refer to those in which NO interacts directly with biological targets, influencing their function. In contrast, indirect reactions involve the formation of molecules derived from NO, such as reactive nitrogen oxide species (RNOS). Consequently, NO produced at low concentrations for short durations by constitutive nitric oxide synthases (eNOS and nNOS) primarily mediates direct effects. On the other hand, indirect effects are more likely to occur in regions where higher local concentrations of NO are maintained over longer periods, typically produced by iNOS. Thus, it is evident that elevated levels of RNOS can lead to conditions characterized by oxidative and nitrosative stress, which can have detrimental effects on cellular function and contribute to disease progression [34]. However, recent evidence has shown that reduced NO bioavailability plays a crucial role in contributing to a low-grade inflammatory state in both vascular and adipose tissues. This condition can lead to serious health issues, including atherosclerosis and insulin resistance [35]. The significant decrease in NO bioavailability may be attributed to several interconnected factors, including lipid peroxidation, oxidative stress, inflammatory responses, and alterations in angiogenesis within the cardiovascular system. Increasingly, researchers and scholars are recognizing these issues as pivotal causes of endothelial injury, highlighting the importance of maintaining NO levels for vascular health and metabolic function [36].

## 4. Carcinogenic Risks of N-Nitrosamines from Dietary Nitrites

Nitrite can also result in the formation of nitroso compounds (NOCs), including N-nitrosamines, which have been shown to have carcinogenic potential in the stomach and intestines. This carcinogenicity is primarily due to their direct interaction with DNA, resulting in harmful processes such as deamination and nitration [37].

Once formed, N-nitrosamines can reach target tissues, such as the stomach, colon, and other parts of the gastrointestinal tract, where they are metabolized by cytochrome P450 enzymes (CYPs). This metabolic activation involves the conversion of N-nitrosamines into highly reactive electrophilic species that can bind covalently to DNA, leading to the alkylation of the DNA bases [38]. The DNA damage caused by these adducts can lead to mutations during DNA replication, as the damaged bases are mispaired with incorrect bases, ultimately promoting genetic instability. This mutagenic process can disrupt normal cell function, leading to the initiation of carcinogenesis. Additionally, the formation of these adducts can also trigger the activation of tumor suppressor genes and oncogenes, contributing to the transformation of normal cells into malignant ones [38].

In 2023, the European Food Safety Authority (EFSA) identified 10 carcinogenic N-nitrosamines that can be found in a variety of food items, including cured meats, processed fish, beer, and other alcoholic and non-alcoholic beverages. These compounds are also present in cheese, soy sauce, oils, processed vegetables, and milk. Additionally, heat treatment methods employed during food preparation can generate and substantially raise the levels of N-nitrosamines in food products, with much of the research concentrating particularly on meat and fish items. However, the formation of N-nitrosamines from nitrites, i.e., potassium nitrite (E249) and sodium nitrite (E250), sometimes used as food additives in meat products, may be a cause of low concern for human health whether additive levels remain within the approved range [39]. This highlights the importance of monitoring nitrite levels and cooking methods to mitigate the potential health risks associated with these harmful compounds [40].

In fact, numerous studies have extensively demonstrated the link between nitrosamines and an increased risk of developing cancer. These investigations have focused on various types of cancer, including those affecting the stomach [41,42], colorectal region [43], bladder [44], skin [45], and other organs [46] (Table 1). Due to their potential carcinogenic risks, there is growing pressure to limit nitrates and nitrites in meat products. However, finding effective substitutes is challenging, as these compounds are crucial for preservation and safety. Food science is working on natural, safer alternatives that can perform similar functions without the risks [47].

In 1989, researchers made a crucial discovery when they found that feeding animals with nitrosamines can lead to the development of cancer in various organs [50]. A few years later, further studies demonstrated that there exists an endogenous pathway for the formation of nitrosamines. This occurs when the inorganic anion nitrite reacts with amines from the diet, laying a crucial foundation for studying the nitrosamine health effects in humans [51]. Once nitrites enter the stomach acidic environment, certain enzymes can catalyze the formation of various NO-related compounds, including S-nitroso, N-nitroso, O-nitroso compounds, and NO itself [35]. N-nitroso compounds readily react with amines to form N-nitrosamines, whereas O-nitroso compounds can interact with phenolic groups, and S-nitroso compounds can bind to protein thiols. Typically, the acidic environment of the stomach favors the formation of S-nitrosothiols (products of S-nitroso compounds) over N-nitrosamines. Meanwhile, the formation of NOCs in the gastrointestinal tract may be largely influenced by the intake of nitrates and nitrites in the diet. Research has demonstrated that S-nitrosothiols can offer beneficial effects on cardiovascular health and metabolic disorders by functioning as relatively stable NO donors. This stability enables them to contribute significantly in a range of physiological processes, further highlighting their potential as therapeutic agents in managing health conditions related to the cardiovascular system and metabolism [52].

Hence, several factors can influence the process of nitrosation. For instance, the acidic environment of the stomach and the presence of iron, particularly in the form of heme iron, can greatly promote this process. Conversely, antioxidants like vitamins C and E can help inhibit nitrosation, potentially reducing its harmful impact. Thus, in acidic conditions, especially when oxidative stress is present, nitrites are converted into reactive nitrogen species (RNOS). The buildup of these species leads to nitrosative stress, which can contribute to the development of both acute and chronic diseases [34].

## 5. Impact of Nitrosative Stress on Cellular Health: Mechanisms, Damage, and Disease Implications

Nitrosative stress is a common physiological condition that arises as a result of the cellular production of RNS, such as peroxynitrite (ONOO^−^). This RNS is generated when NO reacts with superoxide anion, and it can subsequently interact with various biomolecules to form additional RNS. One significant consequence of peroxynitrite is its ability to oxidize tetrahydrobiopterin (BH_4_) into its inactive form, BH_2_. This molecule is a crucial cofactor necessary for the production of NO by eNOS. When the bioavailability of BH_4_ decreases due to oxidation, it causes the uncoupling of eNOS, leading to an increased production of superoxide and a decrease in NO levels. This creates a vicious cycle that exacerbates the reduction in NO bioavailability, further contributing to the overall nitrosative stress within the cellular environment [30].

Nitrosative stress is effectively balanced by the presence of various antioxidant molecules, which are essential for preserving cellular health. When there is an increase in oxidative and nitrosative stress, it typically defines a pathological state in which the body’s antioxidant defenses are insufficient to completely neutralize the ROS and RNS that are formed. This insufficiency can arise due to excessive production of ROS/RNS, a decrease in the body’s antioxidant defenses, or a combination of both factors. The subsequent condition can result in considerable damage to essential cellular components, including nucleic acids, lipids, and proteins. Such damage can significantly impair cell health and function, potentially generating secondary reactive species that further worsen the situation. Ultimately, this cascade of events can cause cell death through mechanisms like necrosis or apoptosis, posing serious risks to overall tissue integrity and organismal health [53]. The oxidative and nitrosative damage to these essential biomolecules, if not controlled or addressed, could potentially play a role in the onset and progression of several diseases. This unchecked damage may disrupt normal cellular functions and pathways, resulting in a series of detrimental effects that could eventually cause significant health complications. RNS can react with proteins and alter their structure with consequences on their function, such as the inhibition of enzymatic and binding activities, increased tendency for aggregation and proteolysis, and changes in immunogenicity [49]. These modifications occur primarily in two forms: the S-nitros(yl)ation of cysteine thiols or the nitration of tyrosine residues. Both reactions are selective and can be affected by specific factors, with the local environment of the residue playing a key role. It has been shown that tyrosine nitration is promoted by the proximity of turn-inducing amino acids such as proline or glycine and a nearby negative charge [54], while the S-nitrosylation of cysteine thiols is driven by the presence of an acid-base consensus motif [55]. An interesting study showed that the presence of hydrophobic pockets within proteins enhances the S-nitrosylation of internal cysteines by accumulating NO and O_2_, which are naturally hydrophobic [56]. Nitrosative stress can also trigger lipid peroxidation that causes changes in the membrane fluidity, resulting in increased tissue permeability and the inactivation of membrane-bound receptors or enzymes. Additionally, the lipid peroxidation process produces a range of relatively stable products that have been shown to readily react with proteins, DNA, and phospholipids, leading to the formation of compounds that contribute to the development of various diseases [48]. The decreased synthesis and/or bioavailability of the vasodilator NO, along with the impairments caused by inflammation, senescence, and oxidative stress, contribute to endothelial dysfunction. This dysfunction is a key factor in the development of various cardiovascular diseases (CVDs), including atherosclerosis [57]. Consequently, due to the elevated levels of RNS, nitrosative stress is identified as a significant characteristic of many acute and chronic diseases [34].

## 6. The Essential Role of Antioxidants in Reducing Oxidative Stress and Supporting Health

Radical molecules, such as RNOS, are produced naturally as byproducts of metabolic processes and serve critical functions in cellular signaling and defense mechanisms. One prominent example is NADPH oxidase, an enzyme complex found in phagocytes like neutrophils and macrophages, which generates superoxide radicals (O_2_^−^) during the respiratory burst. Superoxide anion can then react with NO to form peroxynitrite (ONOO−), a highly reactive and damaging species [58]. This oxidative process is a part of the immune response, where these radicals are used to neutralize and destroy invading pathogens, such as bacteria and fungi. However, the same radicals that are vital for pathogen defense can also be deleterious when their production is uncontrolled or excessive.

Increased levels of RNOS can result in oxidative stress, causing damage to cellular components such as proteins, lipids, and DNA, which can promote inflammation, accelerate aging, and contribute to the onset of various diseases, including cancer, cardiovascular disorders, and neurodegenerative conditions. Therefore, these radical molecules have a dual role: beneficial in moderation for immune defense and signaling, yet potentially harmful when produced in excess or when the body’s antioxidant defenses are insufficient. The balance between their protective and harmful effects relies on the regulation of their production and the efficiency of cellular antioxidant systems. Exposure to free radicals from various sources has led organisms to develop a wide range of defense mechanisms. To prevent oxidative and nitrosative stress, cells actively combat ROS and RNS through a strong antioxidant defense system. This system includes both enzymatic and non-enzymatic antioxidants, which work together to protect the body from the damaging effects of free radicals. Together, these antioxidants are essential for maintaining cellular health and preventing the detrimental consequences of oxidative stress [59].

The different antioxidants are available in a wide range of concentrations throughout body fluids and tissues, and their expression tends to increase significantly when the levels of oxidative and nitrosative stress are elevated. This adaptive response underscores the body’s ability to recognize and react to harmful conditions, thereby enhancing its protective mechanisms against potential cellular damage. As oxidative and nitrosative stress levels rise, the body mobilizes these antioxidants to help restore balance and safeguard overall health. The primary enzymatic antioxidants include superoxide dismutase (SOD), which converts O_2_^.−^ to H_2_O_2_; catalase or peroxidase, which transform H_2_O_2_ to H_2_O; and glutathione peroxidase, which reduces H_2_O_2_, hydroperoxides (R-OOH) and lipid peroxide to H_2_O. The regulation of gene expression for these enzymes is influenced by the state of the individual cell, as endogenous antioxidants play a vital role in maintaining optimal cellular functions and supporting overall health and well-being. However, under certain conditions, endogenous antioxidants may be inadequate, and exogenous antioxidants obtained from diet may be necessary to support optimal cellular function [60]. Certain dietary compounds, which do not directly neutralize free radicals but instead boost the body’s endogenous antioxidant activity, can also be considered antioxidants. These include essential nutrients like vitamins E and C, as well as thiol antioxidants like glutathione, thioredoxin, and lipoic acid. Additionally, carotenoids, natural flavonoids, and other beneficial compounds are also a part of this category. Together, these substances enhance the body’s ability to fight oxidative stress and support overall health [61]. The primary mechanism by which antioxidants neutralize NO is through direct scavenging, forming stable products that are less reactive. Several antioxidants, including ascorbic acid (vitamin C) and polyphenols, have been shown to effectively scavenge NO. For instance, ascorbic acid can reduce NO to nitrite (NO_2_−), a less reactive species [62]. Additionally, flavonoids such as quercetin and catechins can also directly interact with NO, reducing its bioavailability and subsequent reactivity with superoxide to form peroxynitrite (ONOO−), which is a much more damaging species [63]. Ascorbic acid, also known as vitamin C, is one of the most abundant water-soluble antioxidants produced by plants. It is primarily found in the cytosol and chloroplasts, where it directly neutralizes superoxide and hydroxyl radicals. In addition to its direct antioxidant properties, ascorbic acid also serves as a substrate for the redox enzyme ascorbate peroxidase, a function that plays a key role in stress resistance in plants [64]. Vitamin C can also participate in the regeneration of other antioxidant molecules, such as carotenoids and α-tocopherol, linked to membranes [65]. In animal organisms, ascorbate plays a central role in several reactions, including the synthesis of collagen and carnitine, and it is also able to increase the bioavailability of dietary iron. Tocopherols, commonly known as vitamin E, are fat-soluble compounds produced exclusively by organisms capable of photosynthesis. These compounds are found within the lipid bilayers of cellular membranes, with a particularly high concentration in the membranes of chloroplasts [66]. The expression “vitamin E” refers to a group of four natural forms of tocopherols (α, β, γ, and δ), which are further expanded to include four additional forms known as tocotrienols (α, β, γ, and δ). All of these molecules are soluble in lipids and possess strong antioxidant properties. They interact with peroxyl radicals at a faster rate than polyunsaturated fatty acids, effectively interrupting the chain reaction involved in lipid peroxidation [67]. Beyond its antioxidant function, vitamin E may also play a structural role by contributing to the stabilization of cellular membranes. Carotenoids are a class of lipid-soluble antioxidants. Among them, β-carotene is one of the most significant, though they are commonly found in membranes and lipoproteins. Carotenoids are especially effective at neutralizing singlet oxygen and can also scavenge peroxyl radicals under conditions of low oxygen, doing so with an efficiency comparable to, if not greater than, α-tocopherol. Since such conditions are common in many biological tissues, carotenoids contribute to the prevention of lipid peroxidation within the body. Furthermore, β-carotene is the precursor of vitamin A, which functions as an antioxidant, though its effectiveness does not rely on oxygen concentration [68]. They are an essential class of micronutrients in the human diet, and are abundantly found in various bacteria, fungi, algae, and plants. These compounds give plant organs, such as fruits, their red, yellow, and/or orange color [61]. Phenolic compounds are a diverse group of molecules defined by the presence of at least one aromatic ring with one or more hydroxyl groups. Major categories within this class include phenolic acids, flavonoids, and tannins, as well as stilbenes and lignans [69]. These compounds are vital antioxidants in higher plants and algae. Flavonoids belong to the polyphenol superfamily, produced by plants and typically found in stems, leaves, flowers, and fruits, which exhibit colors ranging from yellow to light yellow or white. Plants produce flavonoids in response to various environmental and biological stresses, and these compounds also play a role in regulating growth and development [70]. Flavonoids have attracted considerable interest due to their antioxidant and chelating properties, as well as their potential in preventing chronic diseases. One of the most recognized benefits of flavonoids is their ability to protect against oxidative and nitrosative stress. They can efficiently neutralize peroxyl radicals, inhibit lipid peroxidation, and chelate redox-active metals, thereby preventing the catalytic breakdown of hydrogen peroxide (associated with Fenton chemistry) [71]. Under certain circumstances, however, flavonoids may exhibit prooxidant properties, which are thought to be directly related to the total number of hydroxyl groups they contain. Additionally, flavonoids have been shown to influence cell signaling pathways [72]. Dietary antioxidants, including vitamin C, vitamin E, selenium, and polyphenolic compounds, play an essential role in protecting cells from RNOS-induced damage. Recent studies have shown that the intake of fruits and vegetables rich in polyphenols, such as flavonoids, anthocyanins, and resveratrol, can significantly reduce the levels of RNOS in vivo, thereby enhancing cellular defense mechanisms against oxidative and nitrosative stress [73]. Polyphenols can act as both the direct scavengers of RNOS and modulators of antioxidant enzyme expression, contributing to a reduction in the damaging effects of RNOS [74]. Glutathione is a multifunctional intracellular antioxidant found in plants and abundant in the cytosol, nuclei, and mitochondria. This water-soluble tripeptide, present in all cellular compartments, is vital for the antioxidant defense system. In addition to acting as a cofactor for glutathione peroxidase to neutralize hydrogen peroxide and aiding in the regeneration of ascorbate in its reduced form via the ascorbate–glutathione cycle, glutathione can directly inactivate superoxide, hydroxyl radicals, and singlet oxygen. Glutathione can directly react with ONOO− to form glutathione disulfide (GSSG) and nitroglutathione, thus preventing its harmful effects [75]. Moreover, like ascorbate, glutathione helps regenerate α-tocopherol in its reduced form [76]. While animal products contain only small amounts of antioxidants derived from the plants they consume, whole plant foods have significantly higher concentrations of antioxidants when considering the amount ingested. Additionally, fiber is found exclusively in plant-based foods and is completely absent in animal products. Fruits and vegetables are key elements in modulating oxidative stress levels through a variety of bioactive compounds. Furthermore, herbs and spices have very high Oxygen Radical Absorbance Capacity (ORAC) values, reflecting their exceptional ability to scavenge free radicals. Antioxidants and fiber from whole plant foods are crucial for body’s capacity to maintain a balanced redox state. Fibers, particularly those found in fruits, vegetables, legumes, and whole grains, serve as prebiotics for the gut microbiota. Prebiotics are non-digestible substances that encourage the growth and activity of beneficial gut bacteria. Soluble fiber, in particular, provides nourishment for these microbes, promoting their growth and improving gut health. As fibers move through the digestive system, they are fermented by the gut microbiota, producing short-chain fatty acids (SCFAs) like butyrate, which support intestinal lining health, reduce inflammation, and may enhance immune function. By supporting the growth of beneficial bacteria, fiber helps maintain a balanced microbiome, which is essential for digestion, immune regulation, and overall health [77]. Therefore, incorporating whole plant foods into the diet as a natural form of medicine, while promoting positive lifestyle choices and minimizing the consumption of foods and activities that deplete antioxidant reserves, can significantly help to achieve a balanced redox state. When this balance is maintained, it becomes possible to prevent, improve, or even reverse diseases associated with oxidative stress (Table 1) [78].

## 7. Impact on Nitrate Levels and Antioxidant Status of Selenium Biofortification

Biofortification refers to the process of enhancing the nutritional profile of plants through conventional breeding or genetic modification, aimed at improving health outcomes. In particular, selenium(Se)-biofortification involves enhancing the Se content in plants. Se is an essential micronutrient that boosts the antioxidant properties of food crops. In Finland, a nationwide Se-biofortification initiative was implemented by adding sodium selenate to fertilizers, effectively reducing selenium deficiency across the population. This program has been linked to improved antioxidant status and a decrease in health problems related to Se deficiency [79].

Although the primary goal of Se-biofortification is to address Se deficiency, its direct impact on reducing nitrate levels in plants is minimal. However, the antioxidant properties of Se significantly enhance the overall health benefits of biofortified crops. Research indicates that Se-biofortification improves the bioactive composition and antioxidant capacity of plants, which may contribute to better health outcomes [80]. Se also plays a role in nitrate reduction through various biological processes. Microorganisms capable of reducing both selenate and nitrate have been identified, demonstrating the interconnectedness of these metabolic pathways. For example, a study on Sulfurospirillum barnesii found that cells grown with nitrate exhibited nitrate reduction rates approximately 11 times higher than those grown with selenate. This suggests that nitrate presence can stimulate its own reduction, potentially influencing the nitrogen cycle in the environment [81]. Other studies have shown that Se acts as a powerful antioxidant, reducing cellular damage from free radicals and lowering the risk of heart disease. The Finnish Se-biofortification program led to a significant increase in dietary selenium intake, which correlated with a marked decline in heart disease prevalence. By increasing Se concentrations in crops and improving human Se levels, the program contributed to a notable reduction in heart disease rates [82]. Se has also been shown to lower the risk of certain cancers by reducing DNA damage and strengthening the immune response. In Finland, the Se-biofortification initiative was associated with a reduced incidence of some cancers. Increased Se intake from enriched foods improved antioxidant status, which may have played a role in lowering cancer prevalence [82]. Although studies specifically examining the direct effect of Se-biofortification on nitrate levels in Finnish plants are limited, Se is known to enhance antioxidant activity in crops. This enhancement improves plant health and nutritional quality. Moreover, Se-biofortification has been linked to increased beneficial mineral content and reduced heavy metal accumulation in crops, which indirectly supports the overall health benefits of Se-enriched foods [83].

## 8. Conclusions

Nitrates and nitrites have become two of the most controversial compounds found in food, both naturally occurring and introduced through processing additives. While nitrate ions themselves are not harmful, 5% to 20% of the ingested nitrates can be converted into nitrites by anaerobic bacteria in the gastrointestinal tract, which are more harmful. This conversion into nitrite and its subsequent metabolism into nitrosamines is associated with negative health effects, particularly an increased risk of gastrointestinal cancer. On the other hand, many studies emphasize the benefits of NO generated from nitrate conversion, which may help regulate blood pressure and improve cardiovascular health.

This dual nature of nitrates and nitrites highlights the complexity of their impact on human health, which has led to continued debate and research in the scientific community. The meat industry is particularly associated with the use of nitrates and nitrites, as these compounds play various roles in meat curing, such as preserving color, preventing bacterial growth, and enhancing flavor. Due to their potential carcinogenic properties, there is a growing demand to limit the use of nitrates and nitrites in meat products. However, finding an effective substitute for these compounds in meat processing is challenging due to their multifunctional roles in preservation and safety. As the food industry seeks alternatives, innovations in food science are working to develop natural and safer preservatives that can mimic the roles of nitrates and nitrites without the associated risks.

Interestingly, most nitrates are consumed through vegetables, with significant variations in nitrate content. Leafy greens, such as arugula and spinach, tend to have the highest concentrations of nitrates. Despite the presence of nitrites in these plant-based foods, consuming vegetables remains associated with health benefits and a reduced risk of cardiovascular diseases. These benefits are largely attributed to the antioxidants found in these foods, such as vitamins and polyphenols, which contribute to overall well-being.

Looking to the future, there is significant promise in advancing biofortification techniques to optimize the nutrient content of plants, including their nitrate levels. By targeting the nitrate–nitrite conversion pathways within plants, we could potentially create crops that balance the health benefits of nitrate-derived nitric oxide, while minimizing the formation of harmful nitrosamines. This type of plant-based intervention could provide a safer, more sustainable way to harness the positive effects of nitrates, offering a possible solution to the challenges presented by the consumption of processed meats.

Additionally, another promising area of biofortification is Se enrichment, which has been shown to enhance human health by improving immune function, reducing oxidative stress, and potentially lowering the risk of certain cancers. Se-biofortification involves increasing the selenium content in crops through soil enrichment or plant breeding, resulting in foods that are higher in this essential micronutrient. Given that selenium deficiencies are a global health concern in some regions, biofortified crops could provide a cost-effective, sustainable solution to improve Se intake, especially in populations where soil levels of Se are low. By biofortified crops such as wheat, rice, and legumes with Se, it is possible to enhance the nutritional value of everyday foods, offering a new avenue for preventing Se deficiency-related diseases. This approach could be integrated into broader strategies for enhancing plant nutrition, alongside efforts to optimize nitrate content and other micronutrients, ensuring a more comprehensive approach to public health.

In conclusion, while the debate over nitrates and nitrites continues, there is hope for a future where innovative techniques, such as biofortification, can help create safer, healthier food options. The growing understanding of plant biochemistry and the potential for technological advancements could lead to a new era of food that better supports human health while minimizing the risks associated with certain compounds.

## Figures and Tables

**Table 1:** Differences between animal-based and plant-based dietary nitrates and nitrites: adverse effects and healthy benefits.

Adverse Effect	Reference	Healthy Benefits	Reference
Carcinogenicity due to the formation of NOCs and their direct interaction with DNA	[32,37]	Homeostasis	[30]
Nitrosamine intake contributes to stomach cancer	[41,42]	Cardiovascular system and metabolism	[35]
NOCs association with colorectal cancer	[43]	Immune response modulation	[48]
Red meat consumption and increased risk of bladder cancer	[44]		
RNO involvement in skin cancer	[45]		
Dietary intakes of nitrate and nitrite and their associations with site-specific cancer risks	[46]		
Indirect association of consumption of red meat with added nitrite and nitrate and elevated likelihood of stroke	[33]		
Formation of compounds, i.e., RNOS, leading to conditions of nitrosative and oxidative stress	[30,34]		
Altered immunogenicity due to RNS interactions	[49]		

## Data Availability

No new data were created or analyzed in this study. Data sharing is not applicable to this article.
